# Role of Artificial Intelligence in Human Papillomavirus Status Prediction for Oropharyngeal Cancer: A Scoping Review

**DOI:** 10.3390/cancers16234040

**Published:** 2024-12-02

**Authors:** Andrea Migliorelli, Marianna Manuelli, Andrea Ciorba, Francesco Stomeo, Stefano Pelucchi, Chiara Bianchini

**Affiliations:** ENT & Audiology Unit, Department of Neurosciences, University Hospital of Ferrara, 44100 Ferrara, Italy

**Keywords:** oropharynx, HPV, artificial intelligence, radiomics, machine learning, deep learning

## Abstract

Human papillomavirus (HPV) infection is sexually transmitted and commonly widespread in the head and neck region; however, its role in tumor development and prognosis has only been demonstrated for oropharyngeal squamous cell carcinoma (HPV-OPSCC). The aim of this review is to analyze the results of the most recent literature that has investigated the use of artificial intelligence (AI) as a method for discerning HPV-positive from HPV-negative OPSCC tumors. A review of the literature was performed using PubMed/MEDLINE, EMBASE, and Cochrane Library databases, according to PRISMA for scoping review criteria (from 2017 to July 2024). A total of 15 articles and 4063 patients have been included. Eleven studies analyzed the role of radiomics, and four analyzed the role of AI in determining HPV histological positivity. The results of this scoping review indicate that AI has the potential to play a role in predicting HPV positivity or negativity in OPSCC. Further studies are required to confirm these results.

## Simple Summary

This review elucidates the manner in which artificial intelligence (AI) is transforming the diagnosis and staging of squamous cell carcinomas of the oropharynx (OPSCC). The review examines the potential utilization of AI in discerning the status of human papillomavirus (HPV) in OPSCCs. AI is primarily employed in the analysis of imaging data and the interpretation of histological specimens. While the outcomes are encouraging, they require further validation before they can be adopted in clinical practice.

## 1. Introduction

Human papillomavirus (HPV) infection is sexually transmitted and is a causal factor in the development of several cancers, including head and neck squamous cell carcinoma (HNSCC) [1].

HNSCC represents the seventh most common cancer globally, with an estimated 300,000 deaths per year [2]. It is expected that the incidence of this condition will increase by approximately 30% on a global scale, with the rise in HPV infection representing the primary driver of this trend [3,4].

HPV status (presence or absence of HPV infection in tumor cells) plays an important role in the prognosis of patients with oropharyngeal squamous cell carcinoma (OPSCC). Indeed, patients with HPV-positive OPSCC have superior survival rates compared to those with HPV-negative ones. Moreover, patients with HPV-positive OPSCC tend to exhibit distinct clinical and demographic features compared to those with HPV-negative OPSCC [1].

In consideration of the distinctions in oncogenesis and prognosis, the eighth edition of the AJCC (American Joint Committee on Cancer) categorizes OPSCC tumors as HPV-positive or HPV-negative, resulting in disparate staging between the two groups [5].

The treatment of OPSCC is primarily determined by surgery, radiotherapy, chemotherapy, and immunotherapy. These modalities may be employed individually or in combination.

Given that patients with HPV-positive OPSCC have a more favorable prognosis, recent studies have focused on de-intensifying treatment strategies for this group of patients, with the aim of reducing the adverse effects of treatment while maintaining high survival rates [1].

In recent years, there has been a marked increase in the use of artificial intelligence (AI) in the medical field, leading to significant modifications and enhancements in healthcare models [6].

The principal objective of AI is to enhance and augment human capabilities, facilitating the analysis of vast quantities of data in a relatively short timeframe for healthcare professionals. AI is a field of study that encompasses a number of sub-fields. Two of the most prominent of these are machine learning (ML) and deep learning (DL). ML involves the use of algorithms that allow the program to improve autonomously, while DL learns by using very large numbers of multi-level connected processes and exposing these to a large number of examples [7,8,9].

To date, the primary application of AI in the healthcare sector has been in the analysis of radiological images. Recently, AI, through the utilization of DL and ML models, has also been employed in the domain of head and neck oncology, yielding favorable outcomes. One potential application is the recognition of HPV positivity in patients with OPSCC.

The aim of this review is to analyze the results of recent literature that has investigated the use of AI as a method for discerning HPV-positive from HPV-negative OPSCC tumors.

## 2. Materials and Methods

**Figure 1:**
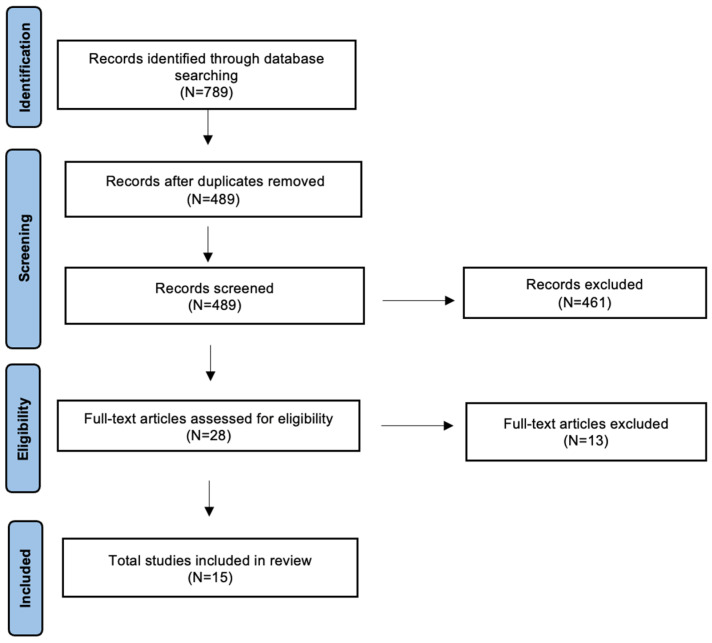
The literature review was performed using PRISMA guidelines for a scoping review.

A detailed review of the English-language literature on AI in HPV-related OPSCC was performed using PubMed/MEDLINE, EMBASE, and Cochrane Library databases. The search period was from 2017 to July 2024, with the aim of selecting the most recent studies. The terms used were “oropharyngeal cancer”, “HPV related oropharyngeal cancer” or “HPV-OPSCC” and “Artificial Intelligence”, “AI”, “Deep Learning”, “DL”, “Machine Learning”, “ML” or “Radiomics”. The search yielded 789 candidate articles. The search was performed according to the “Preferred Reporting Items for Systematic Reviews and Meta-Analyses” (PRISMA) for scoping review guidelines (Figure 1) [10]. The inclusion criteria applied were as follows: (i) publication date after 2017; (ii) studies aiming to use artificial intelligence to distinguish HPV-positive from HPV-negative OPSCC; and (iii) English language. Conference abstracts, case reports, retrospective studies, and publications written in a language different from English have been excluded. Two authors (AM and MM) have independently evaluated all titles, and relevant articles have been individuated according to inclusion/exclusion criteria; a senior author (AC) resolved any disagreements. At the end of the full-text review, 15 articles met the inclusion criteria [11,12,13,14,15,16,17,18,19,20,21,22,23,24,25].

## 3. Results

This scoping review has included 15 articles in total, with 4063 patients analyzed overall. The articles were sourced from a range of geographical regions, with a particular prevalence from Europe and North America. The current application of AI to identify HPV positivity in OPSCCs encompasses two principal areas: (i) the utilization of radiomics in combination with various imaging techniques and (ii) histopathological analysis. These two macro-areas will, therefore, be analyzed separately in the following sections.

### 3.1. HPV and Radiomics

A review of the literature revealed that 11 of the 15 articles addressed the role of radiomics in the identification of HPV-positive OPSCCs [11,12,13,14,15,16,17,18,19,20]. The findings of the review of the application of radiomics are presented in Table 1.

Four articles based their analysis on computed tomography (CT) images, four on magnetic resonance imaging (MRI)-derived images, two on positron emission tomography (PET), and one on PET/CT.

The resulting analysis included 2460 patients with HPV-positive and 1216 patients with HPV-negative cancer. The area under the curve (AUC) values obtained from the various studies ranged between 0.71 and 0.95. The analysis does not identify a single imaging technique as being demonstrably superior to the others.

### 3.2. HPV and Histopathology

The results of the review process about HPV and histopathology are summarized in Table 2. This scoping review has included four articles analyzing the role of AI in determining HPV histopathological positivity in OPSCC [22,23,24,25]. Three of the articles originate from Europe, while the remaining one is from Japan.

Klein et al. [22] obtained an AUC of 0.80 from their model at 40× magnification. This result was compared with that obtained by experienced anatomic pathologists and was found to be superior. Furthermore, Fouard and colleagues found an accuracy of 91% in HPV detection [23]. The two further studies also showed an AUC higher than 0.80, reaching values even higher than 0.90 [24,25].

## 4. Discussion

In this scoping review, we have analyzed the role of AI in predicting HPV positivity in OPSCCs. Our analysis revealed that the primary applications of AI in this field are driven using radiomics and the analysis of histopathological samples.

AI is a technology designed to emulate the cognitive processes of humans, such as learning and problem-solving, using algorithms. The growth in scientific discovery and the integration of computer systems has led to AI becoming a significant tool in healthcare over the past decade [6]. AI has several applications in healthcare, including assisting professionals, educating young doctors, and assisting patients [6,26]. At present, its principal function is to facilitate the analysis of images, whether radiological or histological, by specialists. Its principal strength lies in its capacity for learning and training using DL or machine ML algorithms [6,9]. Furthermore, technological advancement has resulted in the development of language models that acquire their capabilities through the analysis of articles published on the Internet, thereby enabling them to learn from a vast amount of data [27,28]. One of the most extensively researched applications is its role in the field of oncology.

HPV is a DNA virus that infects the skin and mucous membranes. To date, more than 100 different subtypes have been classified. The cervix is the most extensively studied anatomical region with regard to the oncological role of HPV. The high-risk HPV subtypes include 16, 18, 31, 33, 35, 39, 45, 51, 52, 56, 58, 68, 69, and 73 [29]. In the head and neck region, HPV infection also plays a causative role in the development of OPSCC, with an estimated prevalence of 40–60% [30,31]. Of the various HPV subtypes, HPV16 is responsible for 80–90% of HPV-positive OPSCC cases [32]. The most widely used method for diagnosing HPV-positive OPSCC is immunohistochemistry, which detects high expression of the cellular protein p16INK4a. This method is considered in the eighth edition of the AJCC [5].

According to the data from the present review, the majority of the included studies employ p16 as an HPV positivity marker. Nevertheless, the utility of p16 positivity as a marker for patients’ risk stratification is debated, given that single p16 positivity does not necessarily indicate HPV-driven carcinogenesis. Consequently, some studies implement HPV DNA detection by in situ hybridization to evaluate HPV positivity. Two of the studies analyzed this feature by immunohistochemistry and by in situ hybridization [22,24]. Furthermore, Fouad et al. [23] evaluated tissue microarrays stained by in situ hybridization using high-risk HPV genomic probes and a dedicated enzymatic procedure.

HPV-positive OPSCC most frequently affects males aged 40–55 years, with minimal exposure to tobacco and alcohol [32]. These tumors have a significantly more favorable prognosis than HPV-negative OPSCC. HPV-positive OPSCC is more prevalent in North America and Europe [3,4]. This geographical distribution is also corroborated by the provenance of the studies included in our review, as these areas are more frequently affected, and there is greater interest in the scientific field, resulting in a greater number of publications.

It is, therefore, of the utmost importance to analyze the presence or absence of HPV infection in OPSCCs, as this allows the provision of an appropriate treatment plan and prognosis for the patient. Indeed, numerous studies have assessed the potential benefits of reducing the intensity of treatment in HPV-positive OPSCC patients, with the aim of reducing the side effects of therapy and improving quality of life while maintaining similar survival rates [1].

In this context, where the accurate determination of HPV positivity in OPSCCs is of paramount importance, AI can play a pivotal role in assisting radiologists, anatomic pathologists, and oncologists in the staging of these patients. The first application of AI in medical imaging is in the field of histopathology, where it has been shown to achieve diagnostic accuracy in certain cases that is superior to that of human observers. DL is employed in pathological anatomy to facilitate the detection of tumors, determine grading, and search for biomarkers. Consequently, it is also employed in the search for HPV positivity in OPSCC [25]. Slides stained with hematoxylin and eosin are the most commonly used, with the images extracted and subjected to AI models at various magnifications. One of the principal constraints on the utility of DL models for the evaluation of histopathological images is the heterogeneity of the tissue present within a single slide. It is not uncommon for a slide to contain a substantial quantity of non-tumor tissue in addition to tumor tissue [33]. To address this limitation, two main supervisory approaches have been identified in the literature, namely, fully and weakly supervised. The former approach, in contrast to the latter, necessitates additional input from the pathologist, who is required to manually annotate the tumor area on each slide [34].

In 2021, two studies demonstrated that AI can achieve excellent results in the identification of HPV-positive tumors, with an AUC of 0.8 and an accuracy of over 90% [22,23].

Remarkably, both studies employed in situ hybridization techniques for the assessment of HPV positivity, in addition to p16.

Klein et al. [22] employed a training model based on the use of hematoxylin-eosin staining, which yielded promising results. This approach enabled the stratification of patients with a favorable prognosis more effectively than p16 alone or the p16 positive/HPV DNA positive dichotomy. Instead, Fouad et al. [23] used a mathematical morphology-based segmentation algorithm for in situ hybridization, achieving a level of accuracy of over 90%.

Recently, it was demonstrated that the CLAM (clustering-constrained attention-based multiple-instance learning) model, when incorporated with tumor annotation (Annot-CLAM), exceeded 0.9 at a magnification of 20× [25].

The utilization of AI can undoubtedly serve as a valuable asset for anatomic pathologists, potentially alleviating the considerable workload they are currently subjected to and enhancing the precision of their diagnostic capabilities.

Furthermore, the use of HPV-DNA testing with molecular techniques may be limited by factors such as poor tissue quality, sample processing, and results interpretation. In contrast, AI has the potential to provide relatively reliable results in a shorter time frame without being affected by DNA degradation, which can occur due to tissue fixation. Nevertheless, further research is required to evaluate the efficacy and feasibility of the clinical applications. These findings, particularly those concerning p16 and HPV DNA positivity, are very promising.

To date, the diagnostic method for HPV-positive OPSCC has entailed a biopsy and immunohistochemical analysis [5]. However, the oropharynx also encompasses regions that are not readily accessible, such as the base of the tongue, necessitating invasive surgical procedures to obtain the biopsy sample. Consequently, the patient is exposed to potential complications associated with the procedure, such as bleeding [35]. Additionally, the presence of coexisting inflammatory reactions may diminish immunohistochemical sensitivity [36].

A multitude of studies have sought to delineate imaging characteristics that may be predictive of HPV status in OPSCC. However, the considerable subjectivity inherent in image interpretation has rendered this approach largely inapplicable and has precluded the attainment of optimal results [37].

To overcome these limitations, radiomics, which aims to convert images into quantitative data that are independent of the operator, has been employed in recent years. Radiomics is a technique that analyzes large amounts of data and features extracted from imaging [38,39,40]. This approach has also been employed with moderate success in the identification of HPV-positive OPSCC. The analysis of the studies in this field reveals that CT and MRI represent the primary imaging techniques employed in radiomics modeling, with PET following as a distant second. In one of the four studies utilizing CT, sensitivity and specificity values of 75% and 72%, respectively, were identified in predicting HPV positivity, with an AUC of 0.95 [15]. The authors achieved excellent results through the analysis of CT images using a DL on a Convolutional Neural Network (CNN) derived from the C3D pre-trained classification network on video. Both p16 and HPV DNA searches were performed to assess HPV positivity, obtaining information through the OPC-Radiomics and Head–Neck–Radiomics–HN1 datasets.

In subsequent studies, the predictive value and AUC were found to be lower, yet the results remained satisfactory. A total of 31 models have been created aiming to assess the HPV status by means of CT images. The most accurate model was the ‘ensemble subspace discriminant,’ which achieved a success rate of 78.7% [19]. Moreover, the potential of explicit artificial intelligence (XAI) in predicting HPV status based on CT images was recently assessed by training a CNN from scratch using OPC-Radiomics datasets. Subsequently, an XAI algorithm was employed for the evaluation of specific regions within the pre-treatment CTs ROI by an independent institutional database. The results obtained are still not suitable for routine clinical application. Additionally, HPV-positive tumors demonstrated a greater prevalence of intratumoral involvement, in contrast to HPV-negative tumors, which have mostly peripheral involvement [21].

Additionally, MRI yielded noteworthy outcomes in predicting HPV positivity, attaining an AUC of 0.87 [12]. Manual delineation of primary tumor volume was performed using T1W, T2W, T1W3D post-contrast, perfusion, and diffusion-weighted sequences. The images were analyzed using the open-source package PyRadiomics 2.2.0, which revealed a correlation between radiomic features suggestive of a smaller, rounder, more homogeneous, and more regular consistency and HPV positivity.

In 2023, Li et al. [20] demonstrated that integrating a multi-sequence-based image fusion model of the primary tumor and lymph nodes resulted in an AUC of 0.91 for predicting p16 positivity. The authors observed that the radiomic features analyzed (LHH first-order kurtosis, GLSZM size zone non-uniformity normalized, LHL GLSZM size zone non-uniformity normalized, LHH GLCM Id, LHH GLSZM small area emphasis, and LLH GLDM dependence entropy) revealed a homogeneous and regular distribution in HPV-positive tumors. Moreover, PET demonstrated favorable outcomes, albeit with a comparatively diminished impact compared to the other two techniques. HPV-positive tumors have a lower average SUVmax and a more homogeneous uptake than HPV-negative tumors [13,17].

It should be noted that the observation is limited by the small number of studies that have based radiomics on PET images. It is notable that the majority of cases analyzed were HPV-positive, with only a negligible proportion being HPV-negative. This imbalance may potentially represent a limitation for the majority of the studies analyzed.

The technique of radiomics is becoming increasingly prevalent in the staging of OPSCC. It can provide assistance to radiologists or nuclear physicians in the management and interpretation of images. Despite the favorable predictive outcomes of HPV-positive OPSCC, radiomics is not yet a substitute for biopsy and subsequent histopathological analysis.

Major drawbacks of this study are as follows: (i) the limited number of studies currently available in the literature, which has resulted in a restricted scope for this review; and (ii) the heterogeneity of the AI models employed, which has introduced challenges in conducting comparisons.

## 5. Conclusions

The results of this scoping review indicate that AI has the potential to play a role in predicting HPV positivity or negativity in OPSCC. At present, the utilization of AI models for the analysis of histopathological images yields more compelling outcomes than radiomics.

In the future, the advancement of technology, implementation of models, and standardization of protocols may facilitate the integration of AI in the management of patients with OPSCC. This could assist clinicians in managing vast amounts of data, enhance diagnostic accuracy, and facilitate the accurate staging of tumors, enabling the most appropriate therapeutic strategy to be proposed for these patients. Furthermore, radiomics may potentially supplant biopsy in the prediction of HPV positivity in OPSCCs.

The goal of AI in predicting HPV positivity in OPSCCs is (i) to facilitate a precise stratification of patients and (ii) to personalize treatments by minimizing potential side effects and enhancing patients’ quality of life.

## Figures and Tables

**Table 1:** Identified papers on radiomics: objective and major results.

Author (Yrs)	Country	Modality	HPV+/HPV−	HPV Detection	Objective	Major Results
Suh (2020) [11]	South Korea	MRI	48/12	P16 or HPV DNA	ML radiomic classifiers on multiparametric MRI images with DWI to predict HPV status in patients with OPSCC	The logistic regression classifier (0.77 ± 0.12) and the random forest classifier (0.76 ± 0.12) exhibited higher mean AUC values than those demonstrated by the XGBoost classifiers (0.71 ± 0.12). The mean sensitivity and specificity of the logistic regression classifier were 0.71 and 0.72, respectively.
Bos (2020) [12]	The Netherlands	MRI	76/77	P16	Evaluate and compare the ability of clinical variables, MRI-based radiomic features, or a combination of these variables to predict the HPV status of OPSCC	Model performance for the clinical, radiomic, and combined models showed an AUC of 0.794, 0.764, and 0.871, respectively.
Fujima (2020) [13]	USA	PET	70/50	P16	To evaluate the diagnostic accuracy of DL analysis based on PET images to determine HPV status in OPSCC	The DL model showed a sensitivity of 0.83, a specificity of 0.83, a positive predictive value of 0.88, a negative predictive value of 0.77, and a diagnostic accuracy of 0.83. The visual assessment by two radiologists was 0.78, 0.5, 0.7, 0.6, and 0.67 (reader 1) and 0.56, 0.67, 0.71, 0.5, and 0.6 (reader 2). The chi-squared test showed a significant difference between the diagnostic accuracy based on deep learning and that based on the radiologist.
Sohn (2020) [14]	South Korea	MRI	52/10	P16	Investigating the potential of MRI-based radiomic features as a biomarker for predicting the HPV phenotype in patients with OPSCC.	The model yielded an AUC of 0.744 (95% CI, 0.496–0.991) and a sensitivity of 69.2%, specificity of 83.3%, and accuracy of 73.7% using a threshold of 0.94.
Lang (2021) [15]	Germany	CT	290/122	P16 and/or HPV DNA	Assessing DL on CT to predict HPV status in OPSCC	CT-based DL models achieved a sensitivity and specificity of 75% and 72%, respectively, in predicting HPV in OPSCC. The pre-trained 3D video model reached an AUC with a mean value of 0.73 and a corresponding training AUC of 0.95. The 3D network trained from zero achieved an AUC of 0.71; the training AUC was 0.83.
Reiazi (2021) [16]	Canada	CT	824/470	P16	Using radiomics models to assess the impact of the imaging scanner in predicting HPV-OPSCC	The development of a machine learning model for the classification of HPV status from radiological images was found to have a strong dependency on the scanner.
Woo (2022) [17]	South Korea	PET	103/23	P16	To develop and validate a diagnostic ML model for assessing HPV status using PET images and clinical conditions	The combined model of PET and clinical data showed the best performance (AUC = 0.78; 95% confidence interval, 0.46–1), outperforming the PET-only model (AUC = 0.48, *p* = 0.047) and the clinical-only model (AUC = 0.52, *p* = 0.142) in predicting HPV status.
Saikia (2023) [18]	USA	PET/CT	242/82	N/A	Predicting HPV status in OPSCC by means of a multimodal feature fusion architecture based on 3D CNN.	Score of 0.746 *F*1 and an AUC of 0.76, an accuracy value confirming that 74% of HPV-positive patients actually had HPV-related OPSCC.
Fazelpour (2023) [19]	USA	CT	278/115	P16	Prediction of HPV status and prognosis of patients with OPSCC by comprehensive analysis of patient characteristics and staging derived from CT scan	The most accurate of the 31 models developed and listed was ‘ensemble subspace discriminant’ with 78.7% and an AUC of 0.83.
Li (2023) [20]	China	MRI	78/63	P16	Developing an MRI-based radiomic model of the primary tumor and lymph nodes for predicting p16 status in OPSCC	The fusion models outperformed the single models with an AUC in T1WI of 0.80 and in T2WI of 0.74. The models based on multisequence imaging outperformed the single models, T1WI/T2WI, 0.74. Finally, the multisequence-based PT-LN fusion model provided the best classification performance with the highest AUC value of 0.91 for predicting p16 expression.
Fanizzi (2024) [21]	Italy	CT	399/192	P16	Evaluate the preliminary XAI model using CT for the prediction of HPV status in OPCSS	The proposed predictive model demonstrated median AUC and accuracy values of 73.50% and 65.22%, respectively. While these results are encouraging, their current applicability in clinical practice remains limited.

Abbreviation: Yrs: Years; HPV: human papillomavirus; computed tomography; N/A: not available; MRI: magnetic resonance imaging; PET: positron emission tomography; ML: machine learning; DL: deep learning; OPSCC: oropharyngeal squamous cell carcinoma; AUC: area under the curve; CNN: convolutional neural network; PT-LN: primary tumor and lymph node.

**Table 2:** Identified papers on AI and Histopathology.

Author (Yrs)	Country	Magnification	Number of Patients	HPV Detection	Objective	Major Results
Klein (2021) [22]	Germany	40×	273	P16 and HPV DNA	To generate a DL-based HPV prediction score on H&E specimens. The results were compared with those obtained by four experienced pathologists.	The AUC obtained by the DL model was 0.8, higher than the median obtained by the four pathologists of 0.74.
Fouad (2021) [23]	United Kingdom	20×	N/A	ISH	Create an image AI framework to determine HPV status in digitized OPSCC tissue microarray samples.	Experimental results show that the technique provides a classification accuracy of approximately 91% in detecting HPV status.
Wang (2023) [24]	United Kingdom	10×	N/A	p16 and HPV DNA	To propose a new DL pipeline for predicting HPV infection status using only full-screen images of H&E-stained OPSCC sections. To assess whether the Digital HPV score generated from the analyzed slides leads to differences in overall survival and specific disease.	The proposed method achieved an AUC of 0.8371, with a value of 0.8397 in inter-cohort validations, demonstrating better performance than others tested.
Adachi (2024) [25]	Japan	10×, 20×, 40×	114	P16	Use of a weakly supervised CLAM model to predict histopathological features of HPV-positive OPSCC. To improve performance, the model was modified to use the annotated tumor area (Annot-CLAM).	The AUC in predicting p16 expression ranged from 0.802 to 0.834 for CLAM models with different magnifications from the whole tissue area. The mean AUC of the Annot-CLAM models constructed using images at different magnifications ranged from 0.900 to 0.905, an improvement over the results of the CLAM models obtained using whole tumor tissue. The best AUC was obtained with the 20× magnification model.

Abbreviation: Yrs: Years; HPV: human papillomavirus; H&E: hematoxylin and eosin; ISH: in situ hybridization N/A: not available; ML: machine learning; DL: deep learning; OPSCC: oropharyngeal squamous cell carcinoma; AUC: area under the curve; CLAM: clustering-constrained attention-based multiple-instance learning; model; Annot-CLAM: CLAM with tumor annotation.
